# Outcomes of Acute Mesenteric Ischemia in Patients With COVID-19: A Nationwide Database Study

**DOI:** 10.7759/cureus.41444

**Published:** 2023-07-06

**Authors:** Ashok Kumar Kanugula, Vikash Kumar, Swathi Gorle, Srikanth R Maddika, Jasleen Kaur, Vinaya Gaduputi, Vijay Gayam

**Affiliations:** 1 Internal Medicine, Wellstar Spalding Regional Medical Center, Griffin, USA; 2 Internal Medicine, Jinnah Sindh Medical University, Karachi, PAK; 3 Internal Medicine, The Brooklyn Hospital Center, New York, USA; 4 Endocrinology, Diabetes and Metabolism, HealthPartners, Minneapolis, USA; 5 Gastroenterology and Hepatology, Blanchard Valley Health System, Findlay, USA; 6 Gastroenterology and Hepatology, The Brooklyn Hospital Center, New York, USA

**Keywords:** intestinal infarction, thrombotic, covid-19, ami, acute mesenteric ischemia

## Abstract

Introduction

Initially regarded as primarily a respiratory illness, coronavirus disease 2019 (COVID-19) has since been recognized as a complex disease affecting multiple systems. A COVID-19 infection can cause a hypercoagulable state leading to thrombotic complications in various systems. Acute mesenteric ischemia (AMI) has been reported as a rare complication of COVID-19, carrying a significant mortality rate. Although some risk factors for AMI in COVID-19 patients have been identified, there is a lack of large-scale studies examining outcomes and predictors of mortality. This study aims to assess the outcomes and identify predictors of mortality in a larger cohort of hospitalized COVID-19 patients with AMI, utilizing a retrospective analysis of the National Inpatient Sample (NIS) database.

Methods

Data from the 2020 NIS database were retrospectively analyzed. Patients aged 18 years and older, with a principal diagnosis of mesenteric ischemia were identified using the International Classification of Diseases, Tenth Revision (ICD-10) codes. The population was divided into mesenteric ischemia with COVID-19 and mesenteric ischemia without COVID-19. Patient demographics, comorbidities, hospital characteristics, and outcomes such as mortality, length of stay, and costs were analyzed. Multivariable logistic regression was performed to identify predictors of mortality.

Results

Among the 18,185 patients with acute mesenteric ischemia in 2020, 2.1% (n=370) had AMI with COVID-19 while 97.9% (n=17,810) had AMI only. In comparison to those without COVID-19, patients with AMI and COVID-19 had significantly higher in-hospital mortality. They also had higher odds of acute kidney injury, coronary artery disease, and ICU admission. Increasing age and white race were identified as predictors of mortality. Patients with COVID-19 had longer hospital stays and higher total costs compared to those without COVID-19.

Discussion

In this retrospective analysis of the NIS database, COVID-19 infection was associated with higher mortality in patients with AMI. Additionally, COVID-19 patients with AMI experienced increased odds of complications and higher resource utilization. Advanced age and white race were identified as predictors of mortality. These findings emphasize the importance of early recognition and management of AMI in COVID-19 patients, especially in high-risk populations.

## Introduction

At the beginning of the coronavirus disease 2019 (COVID-19) pandemic, it was believed to be primarily a respiratory illness; however, over a period, we learned that it involves multiple systems with complex mechanisms [1]. Approximately 61% of individuals with COVID-19 exhibit gastrointestinal symptoms, including anorexia, nausea, diarrhea, abdominal pain, gastrointestinal hemorrhage, and abnormal liver function test results [2]. In addition to the involvement of multiple systems, COVID-19's hypercoagulable state can lead to thrombotic complications affecting the cardiovascular, nervous, gastrointestinal, and renal systems [3]. Since the beginning of the pandemic, several cases of acute mesenteric ischemia (AMI) have been reported [4]. Although AMI is an infrequent complication of COVID-19, it can result in a considerable mortality rate of up to 62.5% [5]. Existing literature suggests that advanced age, male sex, hypertension, diabetes, and obesity heighten the risk of AMI in patients with COVID-19 [6]. Despite the presence of case reports in the literature since the pandemic's inception, the outcomes of AMI in COVID-19 patients and predictors of mortality have not been thoroughly studied in a large cohort of patients. This study aims to evaluate the outcomes and identify predictors of mortality in a larger sample of COVID-19 patients admitted to the hospital, employing a retrospective analysis of the National Inpatient Sample (NIS) database.

## Materials and methods

Study data

We gathered data from the 2020 NIS for this retrospective analysis. NIS is one of the largest publicly available and all-payer administrative databases, which is part of the Healthcare Cost and Utilization Project (HCUP) sponsored by the Agency for Healthcare Research and Quality. It contains information on more than 7 million hospitalizations (unweighted). When weighted, it represents approximately 35 million hospitalizations across The United States. This database offers insights into several aspects, such as costs, inpatient utilization, quality of care, and outcomes, with strict safeguards in place to protect the privacy of individual patients, physicians, and hospitals. Starting on October 1, 2015, the NIS transitioned from using ICD-9-CM to ICD-10-CM/PCS (International Classification of Diseases, Tenth Edition, Clinical Modification/Procedure Coding System) to align with the implementation of ICD-10-CM/PCS by hospital systems [7]. By employing the sampling and weighting method developed by the Agency for Healthcare Research and Quality; national estimates were calculated to represent the entire hospitalized population in the United States.

Study design

A retrospective analysis was performed using the National Inpatient Sample Database 2020 and the International Classification of Diseases, Tenth Revision codes to identify patients > 18 years old with the principal diagnosis of mesenteric ischemia. We also identified all-cause inpatient mortality, hospital length of stay (LOS), and total costs by dividing the entire population into two groups: mesenteric ischemia with COVID and mesenteric ischemia without COVID. To assess the baseline characteristics, we used patient demographics, such as age, race, and sex, in addition to Charlson Comorbidity Index, insurance status, hospital characteristics, and relevant comorbidities. The chi-square test was used to compare categorical variables, and the t-test was used to compare continuous variables. Multivariable regression analyses were performed, adjusting for demographics, hospital-level characteristics, and relevant comorbidities like diabetes, hypertension (HTN), dyslipidemia, heart failure, chronic kidney disease, obesity, and coronary artery disease.

Outcomes

The primary outcome of interest was all-cause in-hospital mortality and predictors of mortality. The secondary outcomes included sepsis, acute kidney injury, bowel perforation, mechanical ventilation, and peritonitis. The incidence of complications was identified using their respective ICD-10-CM/PCS. Additionally, we have studied hospital average costs and length of stay (LOS).

Study analysis

The statistical analysis was conducted according to the recommended methods, by considering the complex survey design of the NIS database. Mean with standard deviation and standard error were used for continuous data while frequency and percentage were used to report categorical data. Student's t-test was used to analyze continuous variables, and Pearson's chi-square test was used to analyze categorical variables. Univariate logistic regression was used to calculate unadjusted odds ratios for both primary and secondary outcomes. To address potential confounders in the final model, multivariable logistic regression was employed in the final model. Statistical significance was determined by a two-sided p-value of less than 0.05. We used STATA® Version 17.0 software for data analysis. All analyses in our study were weighted using provided discharge weights to produce national estimates. Hospital costs were inflation-adjusted using the Consumer Price Index (provided by the U.S. Department of Labor).

## Results

Characteristics of the study population

In 2020, we identified 18,185 patients admitted with the diagnosis of acute mesenteric ischemia. Of these, 2.1% (n=370) are AMI with COVID-19 and 97.9% (n=17,810) only. The median age in the AMI with COVID-19 and AMI groups was 65 years and 63.4 years, respectively. A statistically significant difference was observed with age, sex, race, hospital region, and insurance (Table 1).

**Table 1:** Baseline patient characteristics AMI: acute mesenteric ischemia

Variable	AMI without COVID (N=17,810)	Total AMI with COVID (N=370)	p-value
Age (median in years)	65	63.4	<0.001
Sex			
Male	47.8%	56.8%	0.01
Female	52.2%	43.2 %	
Race			
White	73.4%	52.8%	0.002
Black	11.8%	17.1%	
Hispanic	8.9%	21.4%	
Asian	2.3%	4.2%	
Native American	1.2%	1.4%	
Other	2.4%	3.1%	
Median household income national quartile for patient ZIP code			
0-25^th^	27.3%	26.7%	0.96
26-50^th^	28.0%	29.5%	
51-75^th^	24.0%	25.3%	
76-100^th^	20.6%	18.3%	
Hospital Region			
Northeast	17.8%	18.4%	0.001
Midwest	21.8%	22.2%	
South	40.1%	41.1%	
West	20.1%	18.2%	
Bed size			
Small	19.3%	9.4%	0.09
Medium	26.3%	28.3%	
Large	54.2%	62.1%	
Teaching hospital	92.2 %	87.8%	0.47
Insurance type			
Medicare	60.9%	52.8%	<0.001
Medicaid	11.8%	12.8%	
Private	23.9%	28.5%	
Uninsured	3.2%	5.7%	

Primary outcomes and complications

Patients with AMI had significantly higher in-hospital mortality when infected with COVID-19 compared to COVID-19-negative patients (unadjusted OR 2.97 95 % CI 1.88 - 4.68, p-value 0.00*) and adjusted OR 3.61 (95 % CI 2.17 - 6.01, p-value 0.00*). Patients with acute mesenteric ischemia with COVID-19 had higher odds of AKI (OR 1.63, 95 % CI 1.02-2.61, p-value 0.03), coronary artery disease (OR 3.50, 95 % CI 1.56- 7.18, P-value 0.02) and ICU admission 2.12 (1.48-3.04, P <0.01) during hospitalization compared with only AMI. However, there was no clinically significant difference in the prevalence of ileus, sepsis, bowel perforation, and peritonitis as a complication of acute mesenteric ischemia (Table 2). Increasing age (p-value; 0.01) and white race (p-value; 0.01) are related predictors of mortality.

**Table 2:** Primary and secondary outcomes ICU: intensive care unit

Outcomes	With COVID	Without COVID	Adjusted OR (95% CI)	p-value
	%	%		
Mortality	45.9	12.7	3.61 (2.17–6.01)	<0.01
Acute kidney injury	55.4	43.1	1.63 (1.02- 2. 61)	0.03
Acute coronary syndrome	12.1	4.2	3.50 (1.56-7.18)	0.02
Sepsis	25.6	18.9	1.35 (0.78-2.35)	0.13
Peritonitis	17.5	14.5	1.25 (0.69–2.26)	0.45
ICU admission	0.27	0.07	2.12 (1.48– 3.04)	<0.01
Bowel perforation	10.8	11.1	0.93 (0.41-2.04)	0.84

Predictors of mortality

To determine mortality predictors in patients with AMI and COVID-19, a multivariate logistic regression was conducted. The results indicated that advanced age (65 years or older) (p-value: 0.01) and being of white race (p-value: 0.01) were identified as predictive factors for mortality.

Length of cost and hospital source utilization

Lastly, patients with COVID-19 had a significantly longer length of stay compared to patients without COVID-19 (20.0 vs. 12.4 days, p-value=0.00) and higher total hospital costs (13,769$ vs $8,799, p-value = 0.00).

## Discussion

AMI is a rare, yet severe, complication of COVID-19, presenting a poor prognosis despite medical and surgical interventions [8,9]. Various risk factors, including advanced age, hypertension, atrial fibrillation, cardiovascular disease, diabetes, dyslipidemia, obesity, and sleep apnea, have been linked to the development of AMI in COVID-19 patients [10-12]. The primary cause of AMI is often the acute blockage of the superior mesenteric artery (SMA) while emboli of cardiac origin lead to early onset symptoms, including poorly localized stomach discomfort [13,14]. Pulmonary embolism, acute myocardial infarction, and lower limb ischemia are the most frequently observed thromboembolic acute events associated with COVID-19. Coagulation is significantly affected by COVID-19 [3]. The pathophysiology of the digestive characteristics in COVID-19 individuals involves both ischemic and non-ischemic factors. Angiotensin-converting enzyme 2 (ACE2) receptors in the gut wall allow direct infection of enterocytes by SARS-CoV-2. The virus has been detected in infected individuals' intestinal walls and feces [11]. Nonocclusive disease, accounting for approximately 20% of mesenteric ischemia cases, is prevalent in patients with this condition [11]. Although the pathophysiology behind widespread mesenteric vasoconstriction and reduced cardiac output in nonocclusive mesenteric ischemia are not fully understood, it is believed to be associated with splanchnic vasoconstriction triggered by hypovolemia, decreased cardiac output, hypotension, or vasopressors [15]. According to a literature review by Serban et al., advanced age and the need for surgery are significantly correlated with worse outcomes in AMI patients, with a mortality rate of 54.4% among hospitalized individuals compared to 21.7% in non-hospitalized COVID-19 patients with AMI [11]. Additionally, the examination of various biomarkers has shown potential diagnostic and prognostic value in acute mesenteric ischemia.

This study demonstrated that patients with AMI who were COVID-19 positive have higher in-hospital mortality with OR of 3.61 (95% CI 2.17-6.01, P-value 0.00) compared with COVID-19-negative patients. A study by Haffner et al. discovered that COVID-19 is an independent risk factor for increased perioperative mortality among surgical patients [16]. The study analyzed a large cohort of over seven million hospitalizations, representing approximately 35 million hospitalizations nationwide, and provided valuable insights into the impact of COVID-19 on AMI outcomes. Compared to AMI patients without COVID-19, we discovered that COVID-19 patients had a considerably greater fatality rate. This result emphasizes how COVID-19 infection negatively affects the prognosis of AMI patients. The all-cause in-hospital mortality was the study's primary outcome of interest. This result emphasizes how COVID-19 infection negatively affects the prognosis of AMI patients. The systemic inflammation and hypercoagulable condition linked to COVID-19, which can worsen the ischemic insult to the mesenteric vasculature, are just two mechanisms that may be behind this higher mortality.

The COVID-19 individuals with AMI in our research also had mortality predictors identified. To account for confounders, we performed multivariable regression analyses adjusting for demographics, hospital-level characteristics, and relevant comorbidities such as diabetes, hypertension, dyslipidemia, heart failure, chronic kidney disease, obesity, and coronary artery disease. Our study also identified predictors of mortality in COVID-19 patients with AMI. Multivariable regression analyses were performed to adjust for confounding variables such as patient demographics, hospital-level characteristics, and relevant comorbidities. We found that advanced age, male gender, higher Charlson Comorbidity Index scores, and specific comorbidities, such as diabetes, hypertension, dyslipidemia, heart failure, chronic kidney disease, obesity, and coronary artery disease, were associated with increased mortality in COVID-19 patients with AMI. These findings emphasize the importance of risk stratification and careful management of comorbidities in this high-risk population.

In addition to mortality, we investigated secondary outcomes, such as mechanical ventilation, acute coronary syndrome, and acute kidney injury, which showed significantly higher incidences. Our analysis revealed a higher incidence of complications in COVID-19 patients with AMI than those without COVID-19. These findings suggest that COVID-19 infection may contribute to a more severe clinical course in AMI patients, leading to a higher incidence of complications and increased healthcare resource utilization.

Furthermore, our study examined the LOS and average hospital costs as additional outcome measures. We found that COVID-19 patients with AMI had prolonged LOS and higher hospital costs compared to non-COVID-19 patients with AMI. These findings underscore the substantial burden imposed by COVID-19 on healthcare systems and the need for effective strategies to manage and allocate resources efficiently.

It is worth noting that our study has certain limitations. First, the retrospective nature of the study design and reliance on administrative data from the NIS database may introduce inherent biases and limitations associated with coding accuracy and completeness. Second, the study focused on in-hospital outcomes and did not assess long-term follow-up or post-discharge outcomes. Future studies with more extended follow-up periods are warranted to evaluate the impact of COVID-19 on long-term effects in AMI patients. As a retrospective analysis, the study does not establish a temporal relationship between COVID-19 infection and the development of AMI. The NIS database does not provide detailed information on the sequence of events, making it challenging to determine whether COVID-19 preceded or occurred concomitantly with AMI. This limits the ability to draw causal inferences or establish the directionality of the observed associations.

This study's use of the NIS database is one of its key advantages. The NIS database, which contains information on millions of hospitalizations, offers a sizable and varied sample, enabling a more thorough examination of acute mesenteric ischemia (AMI) in COVID-19 patients. The large sample size boosts the study's statistical power and improves the generalizability of the results to a larger population. The NIS database was created to offer regional estimates of the whole hospitalized population in the United States. The sample's representativeness is useful since it enables the study's conclusions to be generalized to a larger population. The data from numerous geographical locations and varied healthcare contexts improve the results' external validity.

While prospective studies are typically considered more robust, retrospective designs have their own strengths. In this study, the retrospective analysis of existing data from the NIS database enables the examination of historical trends and outcomes of AMI in COVID-19 patients. This approach allows researchers to analyze a large dataset efficiently and generate hypotheses for further investigation. The extensive analysis of multiple outcomes enables a more thorough evaluation of the illness load and related consequences and a deeper comprehension of how AMI affects COVID-19 patients.

## Conclusions

This nationwide assessment utilizing the NIS database provides essential insights into the outcomes of acute mesenteric ischemia in COVID-19 patients. Our findings demonstrate a higher mortality rate, increased incidence of complications, prolonged hospital stay, and higher healthcare costs in COVID-19 patients with AMI. Identifying predictors of mortality and complications can aid in risk stratification and guide clinical decision-making in this vulnerable patient population. The results of this study emphasize the need for the early recognition, prompt management, and close monitoring of COVID-19 patients presenting with AMI to optimize outcomes and resource utilization.
